# The structure and evolution of story networks

**DOI:** 10.1098/rsos.160071

**Published:** 2016-06-29

**Authors:** Folgert Karsdorp, Antal van den Bosch

**Affiliations:** 1 Meertens Instituut, PO Box 94264, 1090 GG Amsterdam, The Netherlands; 2 Centre for Language Studies, Radboud University, PO Box 9103, 6500 HD Nijmegen, The Netherlands

**Keywords:** network theory, cultural evolution, folktales, children's literature, chain letters

## Abstract

With this study, we advance the understanding about the processes through which stories are retold. A collection of story retellings can be considered as a network of stories, in which links between stories represent pre-textual (or ancestral) relationships. This study provides a mechanistic understanding of the structure and evolution of such story networks: we construct a story network for a large diachronic collection of Dutch literary retellings of *Red Riding Hood*, and compare this network to one derived from a corpus of paper chain letters. In the analysis, we first provide empirical evidence that the formation of these story networks is subject to age-dependent selection processes with a strong lopsidedness towards shorter time-spans between stories and their pre-texts (i.e. ‘young’ story versions are preferred in producing new versions). Subsequently, we systematically compare these findings with and among predictions of various formal models of network growth to determine more precisely which kinds of attractiveness are also at play or might even be preferred as explicatory models. By carefully studying the structure and evolution of the two story networks, then, we show that existing stories are differentially preferred to function as a new version's pre-text given three types of attractiveness: (i) frequency-based and (ii) model-based attractiveness which (iii) decays in time.

## 1. Introduction

In his thought-provoking study *Fairytale in the ancient world*, Graham Anderson quotes the following passage by the Greek geographer Strabo (first century BC/AD), which tells the story of a girl called Rhodopis:

They tell the fabulous story (*mytheuousi*) that while she was bathing, an eagle seized one of her shoes from her maid and brought it to Memphis, and while the king was dispensing justice in the open air, the eagle arrived over his head and threw the shoe into his lap. The king was aroused by the *rythmos* of the sandal and the strangeness of the event, and sent all around the country in search of the woman who wore it. When she was found in Naucratis she was brought up country to Memphis and became the king's wife.

Anderson [1, p. 27]

Does this sound familiar? The ‘seizure of the girl's shoe’, the ‘slipper test’ and the ‘marriage to the prince’ are all motifs that resonate one of the best-known fairy tales in modern times: *Cinderella*.^1^ The *Cinderella* story as we know it today is derived from Charles Perrault's story *Cendrillon* (from *Contes du temps passé avec moralités*, 1697). Perrault's retelling adds various elements to the story, of which the following two are mentioned in the folktale catalogue by Aarne & Thompson: a persecuted heroine (1) and a meeting with the prince in advance of the slipper test (3) [2]. Ever since Perrault published his version of the story, *Cinderella* has been retold to new audiences through a variety of channels: books, picture books, films, advertisements, comics, cartoons and so forth. Yet, these retellings of *Cinderella* do not necessarily derive from Perrault's version. In fact, as Stephens & McCallum state, it is more likely that retellers ‘use intermediate versions—to produce a retelling of a retelling’ [3, p. 4]. These ‘retellings of retellings’, we wish to argue, can be considered as the implicit formation of a network of stories, in which links between stories represent pre-textual relationships.^2^ A story network represents a stream of retellings in which retellers modify and adapt retellings in a gradual and accumulative way.

The aim of this article is to offer new perspectives on the structure and development of such story networks. More specifically, we are interested in the dynamics and mechanisms that underly retellers’ choices for particular story versions to base their retellings on. Certain retellings seem to be more attractive than others, making them more likely candidates for further retelling. Arguably, attractiveness can be defined in two ways: *content-* and *context-based* attractiveness.^3^ *Content-based* attractiveness concerns inherent aspects of a story which increase or decrease its likelihood of being retold. For instance, Charles Perrault's retelling of *Little Red Riding Hood* was highly popular until the Brothers Grimm published their version of *Rothkäpchen* in the nineteenth century. Zipes’ thesis is that the Brothers Grimm ‘virtually dwarfed Perrault's version’ by the end of the nineteenth century, because their emphasis on obedience and good behaviour was a better fit for the emerging Victorian image of the child [7, p. 36]. With *context-based* attractiveness, on the other hand, dispositions for certain stories are not determined by inherent features, but, for example, by social factors, such as popularity or prestige of a particular author. Both content- and context-based attractiveness have received a wealth of attention in literary and folkloristic studies of story transmission [8]. However, despite being suggestive and thought-provoking, informal verbal arguments such as Zipes’ account of *Red Riding Hood*, cannot generate specific predictions which can be quantitatively tested and systematically compared to real-world data.

A more parsimonious explanation for the preference of a reteller for particular story versions is that there are no real ‘motivations’ or selection criteria underlying their choices, or, in other words, that their choice is completely random. A large number of studies in evolutionary anthropology and cultural evolution has shown that social transmission can often be characterized as an *unbiased* process in a neutral model of selection in which changes are reduced to ‘random’ frequency effects of competing cultural traits [9–12]. In the case of story transmission, this would mean that stories with high circulation numbers are more readily available and, in the absence of content- and context-based biases, their attractiveness would be entirely proportional to these numbers. However, if we take enticing accounts of story transmission such as the one by Zipes seriously, it seems unlikely that the selection of a particular story for retelling is entirely frequency-based.

In this study, we wish to depart from the hypothesis that a story's attractiveness for further retelling is merely a ‘random’ frequency effect—or, in other words, is driven by *frequency-based attractiveness*—by systematically investigating the possible influence of other attractiveness factors. First, besides *frequency-based attractiveness*, stories might be differentially preferred given their *temporal attractiveness* (TA), which is a form of context-based attractiveness. For instance, it has been shown for academic citation networks that relatively young research is preferred over older studies and that the probability of being cited decays with time [13–16]. Following Stephens & McCallum, we investigate whether this process equally applies to story networks and whether retellers prefer more recent story versions over older ones in producing a retelling [3]. Second, a story might also be more (or less) attractive because, for example, its author enjoys high esteem. This type of context-based attractiveness we will term *model-based attractiveness*. While each of the three types of attractiveness, i.e. *frequency-based, temporal* and *model-based*, could potentially serve as the sole explicatory factor in story transmission, we suggest that these three kinds of attractiveness interact and collectively impact the choice for particular story versions. Thus, explicatory accounts of story transmission need to account for the interaction between all forces of attractiveness in order to arrive at a more adequate and full explanation of retellers’ preferences for particular story versions.

In order to investigate these issues, the current article aims to contribute to the development of methodologies that allow us to induce micro-evolutionary mechanisms underlying macro-evolutionary developments from historical, population-level data [10,12,17–19]. The first challenge is to develop methods to automatically extract story networks from raw texts that express pre-textual relationships. When such story networks are extracted, we can resort to well-studied concepts and methodologies from network theory to describe their topological and macroscopic properties statistically [20]. In this article, specific attention will be devoted to the degree distributions of story networks, because they provide information about the connections between stories and their pre-texts. Some story versions are used only once to produce a retelling, whereas others serve as pre-textual context for many other stories and could be called ‘story hubs’. The central question is, then, how we can characterize the distribution with which stories are selected as pre-text, and how such distributions come into being. Following previous models of network growth [13–15,21], we investigate a growing network model which combines the three aforementioned kinds of cultural attractiveness. We analyse these forces of attractiveness in isolation as well as the interplay between them and show how their degree distributions behave in relation to those of two empirical story networks.

The main object of study in this article is a collection of Dutch literary *Little Red Riding Hood* retellings, which covers a time-span of more than two centuries. It has been demonstrated that the development of the story about the little girl in red is evolutionary in nature: retellers produce modifications of existing retellings that, in turn, serve as pre-texts for new retellings of the most popular fairy tale of the Western world [22]. Furthermore, it is shown that retellers of *Red Riding Hood* prefer to base their retellings on story versions that are published in close temporal proximity. Yet, as we hypothesized above TA alone cannot explain retellers’ choices for particular retellings from the same time period. The current article seeks to acquire a better understanding of which mechanisms possibly underlie the selection of pre-texts by extracting a story network from the data, and subsequently assessing its structure.

There is, however, a considerable difficulty associated with assessing the validity of the extracted story network and its structural properties, as we lack a ‘ground truth’ of which story served as pre-text for a retelling. For this reason, we made the methodological choice of comparing the structure and development of *Red Riding Hood*'s story network to that of a large collection of paper chain letters. This collection consists of over 500 letters from the twentieth century and represents 100 years of cultural copying. Although chain letters are fundamentally different from fairy tales in many respects, they do make an interesting comparison because of their explicit request to replicate and redistribute the contents of the letter—sometimes to a fixed number of people and often within a particular time window. Crucially, because of this request, we can make at least two predictions about the structure and development of a chain letter network. First, it can be expected that chain letters are connected to pre-texts in close temporal proximity. Second, in a perfect chain (i.e. when all successive recipients of a letter adhere to its request), we can expect a graph structure with a relatively uniform degree distribution, in which all stories exhibit approximately equal degree. These expectations we have of the properties of chain letter networks are confirmed by previous studies that have enhanced the understanding of spreading patterns in Internet chain letter networks [23]. Showing that the extracted chain letter network displays structural and developmental properties that are in accordance with our preconceptions about these properties allows us to partially check for the reliability of our methodology, and hence serves to strengthen our confidence in the validity of conclusions based on the *Red Riding Hood* network and its extracted properties.

The remainder of this article is structured as follows. We begin with a description of the two data collections used in this article (§2). After having presented the data collections, we proceed in §3 with a detailed account of the computational and statistical methods used to construct and analyse story networks. In §4, then, we analyse the structure of the two story networks and compare them to those of a model of network growth. The final section offers a discussion about the main findings of this article.

## 2. Data collections

### 2.1. Red riding hood

‘… there once was a little village girl, the prettiest who was ever seen, and the most famous in children's stories …’^4^ Who does not know the story of *Little Red Riding Hood*? As Jack Zipes writes in his seminal book, *The trials and tribulations of Little Red Riding Hood*, it is ‘the most popular and certainly the most provocative fairy tale in the Western world’ [7, p. 343]. Ever since the first literary version of the tale, *Le petit Chaperon rouge*, written by Charles Perrault (from *Contes du temps passé avec moralités*, 1697), the story has been retold, reinterpreted, reconfigured and recontextualized countless times all around the world. Perrault's story, dedicated to Louis XIV's niece Elisabeth Charlotte d’Orleans, is to be read as a parable ‘that warns young ladies to be aware of suave and debonair two-legged wolves who would sweet-talk their way into their beds and ruin their reputations’ [25, p. 33]. The story with its ‘rapacious wolf’ [7] was reworked by the Brothers Grimm in a number of editions of their collection *Kinder- und Hausmärchen* to fit the emerging Victorian image of the child in the nineteenth century [7]. Among the most striking changes made by the Brothers Grimm are (i) the addition of a rescue scene of Rothkäpchen by a hunter (or some other father figure) and (ii) the emphasis on showing obedience and good behaviour.

The first more experimental retellings of the story appeared after World War I, a trend which came to full growth after World War II when the story branched off in many directions. Zipes distinguishes three major branches: (1) retellings in which Little Red Riding Hood becomes increasingly independent, (2) versions that tell the wolf's version of the story and (3) stories that stand out with respect to their aesthetic experimentation [7]. The experimental retelling written by Ivo de Wijs with illustrations by Alfons van Heusden [26] serves as a wonderful example of the third branch as it diverges radically from the tradition, both in form and in content. The story is part of a collection entitled *Roodkapje en de zeven geitjes* (Red Riding Hood and the Seven Kids). This rather unusual juxtaposition of characters from two famous fairy tales serves to emphasize the bricolage nature of the stories to come. Each story in the book tells three different versions of a story. On each page, supposedly aligned fragments of the three versions are placed side by side in three columns, each with its own distinctive typography. The reader is supposed to read all three columns before moving on to the next page. The following fragment serves to illustrate some of the radical changes the story has undergone:

Once upon a time there was a little girl called Gretel. Well, her name was Gretel, but she was called Little Red Cap because of her flaming red hair. Little Red Cap lived with her grandmother in an old tower out in the woods. One day grandmother went to the city to buy biscuits and lemonade—and a cough medicine because Little Red Cap wasn’t feeling well. ‘Come back soon, grandmother,’ said Little Red Cap, ‘walk briskly!’ And grandmother went on her way.

**Figure 1.**
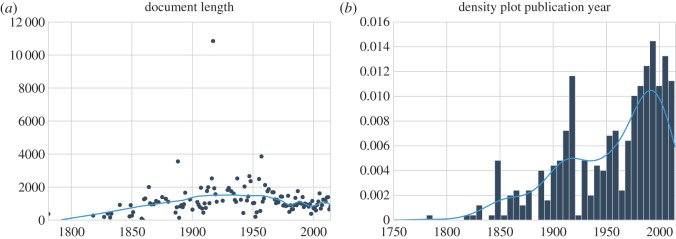
Diachronic visualization of some general statistics about the *Red Riding Hood* corpus. Plot (*a*) visualizes the story length (expressed in word forms), and (*b*) shows a kernel density plot of the number of stories attested per year.

We make use of a diachronic collection of Dutch literary *Little Red Riding Hood* retellings which covers a time-span of more than two hundred years [27].^5^ This collection consists of 427 versions of *Red Riding Hood*. The oldest version dates from the late eighteenth century and the youngest from 2015. The stories were tokenized using the tokenizer Ucto, configured for the Dutch language.^6^ The total number of word forms (including punctuation) in the tokenized corpus amounts to 493 169 of which 10 683 occur uniquely. Each year in the collection is represented by three stories on average. The average story contains about 1155 words. As can be observed from figure 1*a*, the story length has remained stable over time. Figure 1*b* shows the density distribution of publications per year, which is rather skewed towards more recently published retellings (cf. [22]).

### 2.2. Chain letters

In this study, we make use of a rich chain letter corpus collected by Daniel Vanarsdale [28]. His online *Paper Chain Letter Archive* [29] contains over 900 letters, most of which have been transcribed from physical letters. In Vanarsdale's definition, chain letters are letters that explicitly ask the recipients to copy their contents and redistribute them to a (sometimes explicitly given) number of successive recipients. Some chain letters explicitly ask the recipients to make modifications to the letters, for example, by adding their name to the existing list of recipients.

Vanarsdale classifies his collection of chain letters into nine categories. In this study, we investigate the development of the largest category, Luck chain letters. The Luck chain letter is generally believed to be derived from the ‘Himmelsbrief’ (Letter from Heaven) [28]. The earliest attestations of the Himmelsbrief date from the seventeenth century. The letters are supposedly derived from a mysterious letter written in golden ink by Jesus and was delivered to the Earth by the archangel Gabriel [30]. The letters generally warn against sin, contain prayers and encourage doing what is right according to Christian beliefs. The most characteristic feature of these letters is their demand to make one or more copies of the letter. The recipient is warned that if (s)he does not believe in the contents of the letter and refuses to follow what it teaches, (s)he ‘will be punished in eternity, and I [Jesus] shall demand your many sins on Judgment Day, and you will have to answer to me for them’ [30, p. 64]. Vanarsdale classifies the Luck chain letters of the twentieth century into 12 distinctive chronological types, which he describes as the ‘mainline—a century long stream of copying’. Most types display clear influences of prior letter types. The latest letter type ‘Death-Lottery’ which predominantly circulated from 1973 until 2005, for example, is a reversal of the ‘Lottery-Death’ letter type. In these later examples of the Luck chain letter, greater emphasis is put on superstitious beliefs and on possible negative effects of breaking the chain. Reflecting modern times, the number of requested copies increases in these letters, as well as the amounts of money people might receive if they obey the letters’ orders. Another key characteristic of these younger letters is the postscript ‘It works’. Vanarsdale observes that after the first attestation of this postscript in 1979, in a few years time all succeeding letters include it.

**Figure 2.**
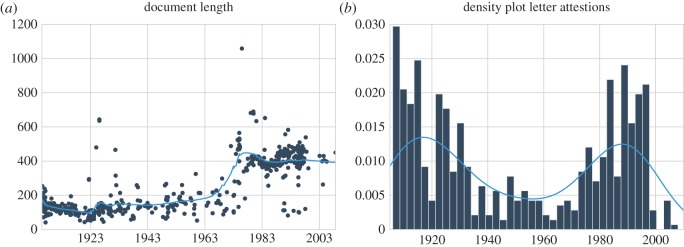
Diachronic visualization of some general statistics about the Luck chain letter corpus. Plot (*a*) visualizes the letter length (expressed in word forms), and (*b*) shows a kernel density plot of the number of letters attested per year.

For this study, we constructed a full-text version of the Luck chain letter collection [27]. The total number of letters in our version of the collection amounts to 554. The oldest letters stem from 1906 and the youngest from 2008. Each year in the collection is represented by six letters on average. We tokenized the collection using the tokenizer of the *Natural Language Toolkit* [31]. The total number of words amounts to 134 589 (including punctuation). In their lowercased form, 5256 of these word forms occur uniquely. Letters consist of 243 word forms on average. In figure 2, we visualize some general statistics of the corpus. Figure 2*a* visualizes how the letter length changes over time. We can observe that until the 1980s the letter length has remained generally stable over time. Around 1980, the letters suddenly become significantly longer, which might reflect some severe changes to the tradition that require further investigation. Figure 2*b* shows the density with which letters have been attested each year. Most letters stem from either the beginning or from the last two decades of the twentieth century.

## 3. Story network construction

Story networks consist of stories and links between stories that represent pre-textual relationships. We make the simplifying assumption that stories that are more similar to each other are more likely to stand in a pre-textual relationship than stories that are more distant. In the following, we discuss a vocabulary-based representation of stories, the well-known ‘bag-of-words’ representation, which is suitable for computational methods of discovering textual similarity. We continue with presenting a clustering algorithm that aims to bootstrap pre-textual relationships between stories from raw collections of texts. Subsequently, we describe how story networks are constructed on the basis of the output of this clustering procedure. Finally, we present network-theoretic statistics to detect and describe pre-textual relationship characteristics of story networks.

### 3.1. Bag-of-words models

Bag-of-words models have proved to be invaluable for numerous computational approaches to textual data, such as text classification, textual information retrieval and textual stylometry. Bag-of-words models make the assumption that maintaining word order is unnecessary in determining the relationship between texts. Obviously, this is a crude simplification, yet there is a surprising number of applications in which word order adds barely any additional information. Given a corpus *C* consisting of a vocabulary *V* (i.e. word types), bag-of-words models represent documents as histograms over the vocabulary, that is, as vectors of occurrence counts of words. For a document *d*, the vector representation **w**^(*d*)^ is given by (*w*_1_,*w*_2_,…,*w*_*V*_), where *w*_*i*_ represents the occurrence count of word *i* in document *d*. To weight the word frequency vectors for term importance, we make use of the well-known ‘term frequency-inverse document frequency’ (tf-idf) weighting scheme, which weights the frequency of words in a document (tf) against the background of how many documents in the collection contain those words (idf) [32]. Using these weighted vector representations, the similarity (or distance) between two stories can be assessed by means of a pairwise comparison of their vector values. We choose to use the cosine dissimilarity measure to express the distance between two stories in terms of their weighted vector representations.

### 3.2. Bootstrapping pre-textual relationships

Given a story, how can we identify a set of potential pre-texts *P* from a collection of stories *C*? Using the story representation discussed in the previous section, we can compute the distance between a story *i* attested at time *t*_*i*_ and all potential pre-texts. We consider stories to be potential pre-texts if and only if they are attested prior to *t*_*i*_, that is *P*={*j* | ∀*j*∈{1,2,…,*C*}∧*t*_*j*_≤*t*_*i*_∧*i*≠*j*}, where *C* represents the complete set of stories in the collection. This set of potential pre-texts can then be sorted in ascending order to obtain a ranking in which the top items represent the most likely pre-texts of story *i*.

We can apply a cutoff to these rankings to assign to each story its *k* most likely pre-texts. At a cutoff of *k*=1, we only take into consideration the most similar (or least distant) text, whereas at *k*=|*P*| the complete set of potential pre-texts is taken into account. Unfortunately, it is not straightforward to define the value *k* in a non-arbitrary way. A more fundamental problem of this approach, however, is the possibility that the set of potential pre-texts does not contain an actual pre-text in the first place. In the most extreme case, the most similar pre-text (*k*=1) is maximally distant from the story under consideration. A possible way to overcome this problem is to define a threshold value at which stories are considered to be too distant to be related. The exact value of this threshold, however, is sensitive to a multitude of factors, such as the corpus under investigation, its feature representation and the distance metric used.

The problem we face is essentially an open-set problem: given a set of potential pre-texts, can we decide which of them are similar enough to be considered actual pre-texts of a particular story? This includes the possibility that *none* of the potential pre-texts are similar enough. The problem as we formulate it here is closely related to the *many candidates* problem in the context of authorship verification [33]. In that problem, the goal is to assess whether the author of some anonymous document is someone among a set of candidate authors, or a yet unknown author [33]. To overcome the sensitive practice of defining ranking cutoff values or distance thresholds, we describe a procedure that allows us to exclude potential pre-texts from consideration in a more robust and less arbitrary way. The procedure draws inspiration from resampling methods [34] and methods proposed to tackle the author verification problem [33].

The general idea behind the procedure is to assess the rankings of pre-texts for their robustness by approaching the data from various angles for a large number of trials. If we compute the distances between a story and its potential pre-texts on the basis of a slightly modified feature space (i.e. we leave out a small number of words from the bag-of-words representation), we want the resulting rankings to be consistent with those of the unmodified feature space. We repeat this process and in each trial we compute the distances between each story and its potential pre-texts on the basis of a random sample (e.g. 50%) of the complete feature space. Each trial potentially produces slightly (or very) different rankings. We select from each trial the *k*=1 nearest-neighbour for each story and compute the fraction of trials *μ* that this potential pre-text was identified as the most similar (or least distant) story. The result of the procedure is a ranking of potential pre-texts for each story *i* in which the positions represent the consistency with which pre-texts have been identified as the nearest-neighbour of *i* over all trials. Some stories unequivocally select the same nearest-neighbour over all trials (*μ*=1), whereas for others the selection is more ambiguous, e.g. the most consistent selection does not exceed 20% of the trials (*μ*=0.2). Depending on how conservative one wants the selection to be, a fixed cutoff value can be used that expresses the lower bounds of the fraction of trials with which a pre-text has to selected as the nearest-neighbour of a story. At *μ*=0, all potential pre-texts are selected, whereas 0.5<*μ*≤1 maximally returns one nearest-neighbour. In the experiments below, we set *μ* to the rather conservative value of 0.5. We will refer to the procedure as *Bootstrap Neighbor Clustering*.

### 3.3. Story network construction and statistical methods

Given a bootstrapped set of pre-texts *A* for story *i*, we represent each pre-text selection as a link using the following notation: i→j where *j* refers to one of the pre-texts in *A*.^7^ In network theory, these links are called directed edges between pairs of vertices. Consider the following set of stories:

3.1 V={1,2,3,4,5},

and the set of pairs of stories and pre-texts, represented by

3.2 E={3→2,4→3,5→3}.

Using *V* and *E*, we create a graph *G*=(*V*,*E*). Note that in this hypothetical example, story 1 is a singleton story for which no decisive pre-text could be determined. The number of incoming edges (i.e. the number of stories that select a particular pre-text) is called *in-degree*. We use the notation *d*_in_(*i*) to refer to the in-degree of node *i*. *Pr*(*d*_in_), then, represents the probability that a randomly chosen node from the network has a particular in-degree.

A common approach to characterize degree distributions is to fit the parameters of a probability density function. Many real-world networks such as the Word Wide Web network and (scientific) citation networks display a degree distribution with a so-called ‘heavy tail’ in which a large proportion of the nodes have a small degree and only a few yet significant number of nodes are connected to many nodes [20]. In some networks, the degree of the nodes decays exponentially, whereas in others it follows a power law:

3.3 $$\mathrm{Pr}(d)\approx d^{-\alpha },$$

where values of the parameter *α* typically fall in the range 1.5≤*α*≤3 [20], table II. Larger values lead to a faster decay in the probability of nodes with a high degree. Given that two degree distributions follow a power law, we can compare the exponents of their distributions. However, as Clauset *et al.* [35] have shown, many empirical distributions that appear to follow a power law are in fact better described using other heavy-tail distributions such as the lognormal distribution. In this article, we apply the techniques developed by Clauset *et al.* [35] to characterize the degree distributions of story networks.

Heavy tails of degree distributions represent an uneven spread of edges among nodes. This inequality can be represented using a Lorenz curve which was developed by the economist Max Lorenz for representing wealth inequality in a population. The curve displays the fraction of wealth held by the richest fraction of people in a population. Applied to network degree distributions, the curves represent what fraction of edges is held by what fraction of nodes. If all nodes in a network are connected by the same number of edges, the curve forms a straight line, representing total equality. In the situation where a small fraction of nodes holds a large fraction of edges, the curve displays a steep increase, indicating that the edges are spread unevenly among the nodes.

The degree of inequality observed in Lorenz curves can be conveniently summarized using the Gini coefficient *G*. This coefficient can be defined as twice the area between the equidistribution and an observed Lorenz curve. *G* falls in the range 0≤*G*≤1 where larger values indicate a higher degree of inequality. A crucial advantage of the Gini coefficient is that it allows us to compare networks of different average degrees and sizes [36].

## 4. Story network analysis

**Figure 3.**
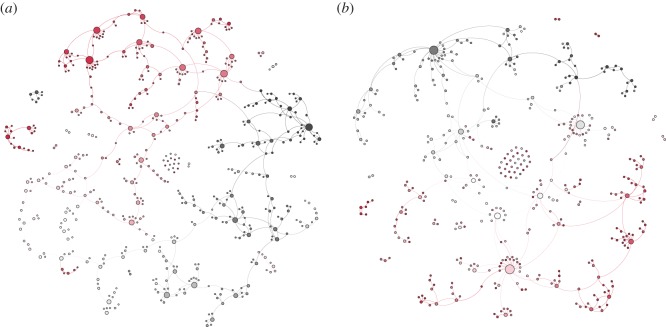
Story networks of the collection of chain letters (*a*) and *Red Riding Hood* retellings (*b*). Nodes represent individual stories and edges represent pre-textual relationships between stories. The colour gradient (from black via white to red) represents the age of each story.

We apply the *Bootstrap Neighbor Clustering* procedure to construct story networks for the collection of *Red Riding Hood* retellings and the collection of chain letters. In figure 3, we provide a graphical visualization of the two networks. The *Red Riding Hood* network consists of *n*=427 nodes and *m*=439 edges. The chain letter network consists of *n*=554 nodes and *m*=620 edges. The colour gradient (from black via white to red) in the two networks represents the age of each story. Two important observations can be made from visually inspecting the two networks. First, let us consider the chain letter network. As indicated in the Introduction and Data sections, chain letters are explicitly designed to be replicated and redistributed within a short period of time. To confirm the reliability of our methodology, then, the story network extracted by the *Bootstrap Neighbor Clustering* procedure has to be in accordance with some of our preconceptions about the structure of chain letter story networks. The visualization in figure 3 indicates that this appears to be the case: most stories in the network are connected to other stories with a similar colour shade, which means that, in the chain letter network, stories predominantly select stories of approximately the same age as potential pre-texts. Interestingly, the second (*Red Riding Hood*) network exhibits a similar pattern, with each story showing a clear preference to select pre-texts with a similar colour shade (i.e. close temporal proximity). Second, it can be observed that the network derived from *Red Riding Hood* retellings has a structure with a few hubs, i.e. where three or four stories function as pre-text for a large number of stories (which is represented by the size of the nodes in the networks). The chain letter network, on the other hand, displays a more uniform distribution over which stories function as pre-text, which also is in accordance with our expectations about the structure of chain letter networks (cf. §1).

In what follows, we will statistically characterize these two observations by carefully studying the in-degree distributions of the two story networks. Accurately characterizing the in-degree distribution of the two networks is a fundamental prerequisite to understand which models of network growth potentially underlie the evolution of the two story networks. A power-law characterization of the in-degree distribution, for example, might be accounted for by models of network growth such as the preferential attachment (PA) model (cf. §4b). Subsequently, we will study the development of the two story networks over time. We compare four models of network growth and analyse their in-degree distributions in relation to those of the two story networks.

### 4.1. In-degree distribution analysis

**Figure 4.**
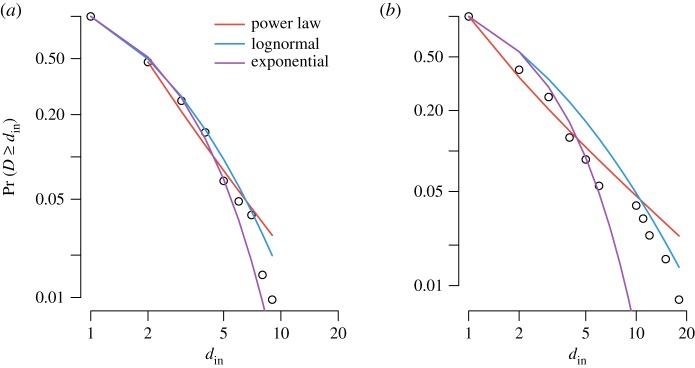
Complementary cumulative in-degree distributions of the two story networks: (*a*) chain letters; (*b*) *Red Riding Hood*. The plot provides for both distributions the best fit of a power law, lognormal and exponential model. The power law and lognormal models both fit the empirical in-degree distribution of *Red Riding Hood* well. The chain letter in-degree distribution is best described by means of an exponential model.

In figure 4, we plot the cumulative complementary distribution function of the in-degree distributions of the story networks on doubly logarithmic axes. The plots express the probability of stories with at least in-degree *d*_in_, which is denoted as *Pr*(*D*≥*d*_in_), where *D* represents a random variable drawn from the distribution. Especially for the in-degree distribution of *Red Riding Hood*, the plots clearly show that the vast majority of stories have a small in-degree and that only a few stories are selected as pre-text by a large(r) number of stories.

To obtain a better understanding of the distributions, we perform the rigorous statistical procedure as described by Clauset *et al.* to detect power-law behaviour in distributions [35]. In many real-world datasets, the power-law property only applies to the tail of the distribution, i.e. for values greater than some minimum value *d*_min_. The method proposed by Clauset *et al.* [35] estimates a minimum value *d*_min_ by comparing the empirical distribution to the theoretical cumulative distribution function. The method aims to optimize the Kolmogorov–Smirnov statistic *D* by choosing *d*_*i*_ as the value for *d*_min_ for which *D* is the smallest. Clauset *et al.* suggest to test whether a dataset actually follows a power law using a goodness-of-fit test and a bootstrapping procedure [35]. The *p*-value resulting from this test answers the question whether possible differences between the empirical data and the model are significant or not. If *p*≃0, then the model cannot be deemed a plausible fit and other distributions are more appropriate.

At a significance level of 95%, we cannot reject the hypothesis that the in-degree distribution of the *Red Riding Hood* network is generated by a power-law model: *D*=0.048, *p*>0.07. By contrast, the power-law model is not a good fit for the in-degree distribution of the chain letter network (*D*=0.09, *p*=0.001). Another way to test for power-law behaviour is to directly compare a power-law model to another model, such as a lognormal model via a *likelihood ratio test* *R* [35]. Using the likelihood ratio test, we can assess whether the in-degree distributions are more appropriately described by means of a lognormal or exponential model. The lognormal model fits the chain letter distribution significantly better than the power-law model for the full range of in-degree values (*TS*=−4.05,*p*<0.0001). The next step is to compare the lognormal model to an exponential model. The test results are indecisive as to whether a lognormal model is more appropriate than an exponential model (*R*=0.079,*p*>0.9). However, since a lognormal function has more parameters than an exponential function, it seems reasonable, for reasons of parsimony, to characterize the chain letter in-degree distribution as exponential. Although a lognormal model appears to fit *Red Riding Hood*'s in-degree distribution slightly better than a power-law function, the test results suggest that the two models do not perform significantly differently (*R*=−1.52,*p*>0.1).

**Figure 5.**
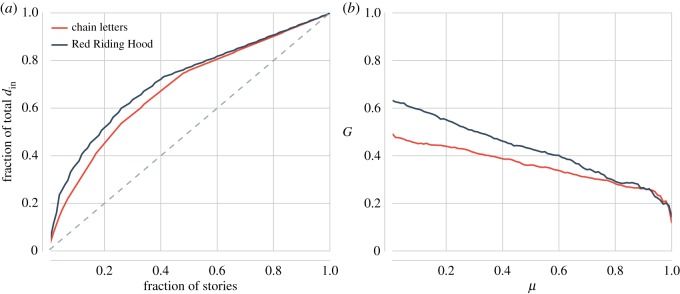
Plot (*a*) displays the Lorenz curves of the chain letter and *Red Riding Hood* story network. The grey dashed line represents the equidistribution. Plot (*b*) shows the Gini coefficient *G* of the in-degree distributions for story networks constructed using *μ* in the range 0.1≤*μ*≤1. It can be observed that the value of *G* remains relatively stable as we increase the value of *μ*.

In figure 5*a*, we show the Lorenz curves of the two in-degree distributions. The summarizing Gini coefficients of these curves are *G*=0.36 for the chain letter network and *G*=0.43 for *Red Riding Hood*. Compared to their random counterparts, the story networks exhibit a greater degree of inequality with respect to their in-degree distributions (chain letters: *G*_random_=0.3; *Red Riding Hood*: *G*_random_=0.28).^8^ The degree of inequality is especially large in the case of *Red Riding Hood*, suggesting that relatively few stories function as pre-text for many other stories. In figure 5*b*, we visualize the Gini coefficients *G* for story networks that were constructed with 0.1≤*μ*≤1. It can observed that even for high values of *μ*, the in-degree distributions display a relatively uneven spread of edges among nodes (i.e. stories). This indicates that the ‘hubness’ of the networks is a stable characteristic and not too much the result of cherry picking a particular value of *μ*.

### 4.2. Story network evolution

In the previous section, we have shown that the two studied story networks display distinct in-degree distributions. The chain letter network exhibits an in-degree distribution which decays exponentially. The *Red Riding Hood* network, by contrast, exhibits a heavy-tail in-degree distribution that fits a power law or lognormal model reasonably well. Retellings of *Red Riding Hood* preferentially link to a small number of stories that are pre-texts of many other retellings. In this section, we turn to the central question of how these distributions come into being. By carefully studying and comparing the structure and evolution of the two story networks to formal models of network growth, we provide empirical evidence for three major conclusions regarding the formation of story networks. First, we provide empirical evidence that stories preferentially select stories in close temporal proximity, which is indicated by a strong lopsidedness towards smaller time-spans in the time-span distributions of stories and their selected pre-texts. Second, we show that the in-degree distribution of the *Red Riding Hood* network is significantly correlated with the age of stories, suggesting that retellings of *Red Riding Hood* are affected by a mechanism of PA in which slightly older versions are preferred to be selected as pre-text(s) in producing a new version. Finally, we show that stories have individual attractiveness values that lessen over time.

The PA model has proved to be a reliable model to account for heavy-tail degree distributions observed in real-world networks. The model was invented by Derek de Solla Price in the context of citation networks [15] and simplified and generalized by Albert-László Barabási and Réka Albert to account for undirected networks [21]. The algorithm generates these networks using an attachment mechanism in which new nodes preferentially link to existing nodes with high (in-)degree. The mechanism of PA predicts that the probability of creating a new link between node *i* and *j* is proportional to the number of existing connections of *j*.

**Figure 6.**
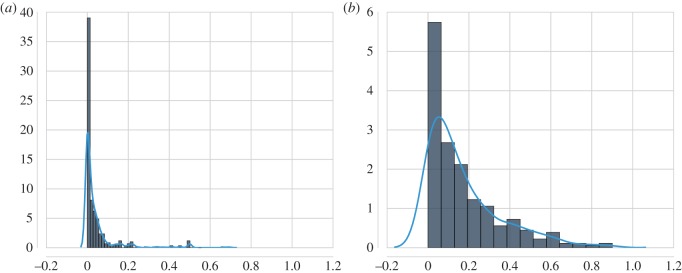
Kernel density plots of time-span distributions. The plots display the distributions over time-spans for (*a*) the chain letter network and (*b*) the network induced for *Red Riding Hood*. As can observed, both time-span distributions display a strong lopsidedness towards smaller time-spans, which indicates a preference for pre-texts in close temporal proximity.

The consequence of the PA mechanism is that nodes that enter a network first will attract more links early on, and will continue to do so. As an explanatory model for story networks, the PA model predicts that stories preferentially select old(er) stories as pre-text rather than new(er) stories. To test this hypothesis, we evaluate for each pair of story and pre-text the time elapsed between their publication dates. In figure 6, we plot kernel density plots of the time-span distribution for the chain letter network and the *Red Riding Hood* network. The time-spans have been normalized to 0≤*τ*≤1, where *τ* represents the normalized time-span between a story and a pre-text. Both plots display strong lopsidedness towards smaller time-spans. Chain letters display an even stronger preference for pre-texts in close temporal proximity (median $\hat{\tau }=0.01$) than the retellings of *Red Riding Hood* (median $\hat{\tau }=0.11$). Both time-span distributions run counter to the prediction of the PA model that stories are predominantly connected to old(er) stories.

**Figure 7.**
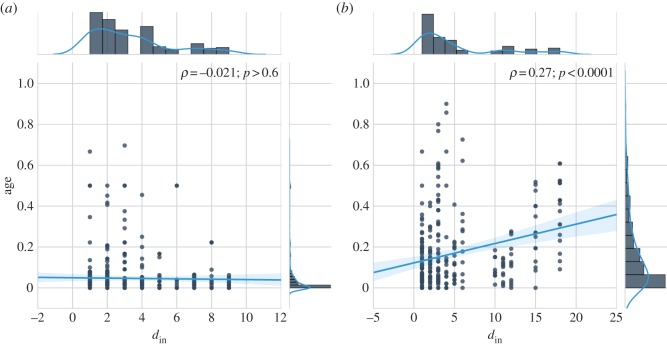
Correlation between in-degree and age. The two plots display the correlation between the age of a story and its in-degree for both (*a*) the chain letter network and (*b*) the *Red Riding Hood* network. The chain letter in-degree distribution does not significantly correlate with the age of the letters (*ρ*=−0.02,*p*> 0.6). The in-degree distribution of the network of *Red Riding Hood* is significantly correlated with age (*ρ*=0.27,*p*<0.0001).

To obtain more insight into the relationship between the in-degree and time-span distributions of the story networks, we test in figure 7 for the correlation between in-degree and age. As can be observed from figure 7*a* and confirmed by a Pearson *ρ* correlation test, there is no evidence that the in-degree distribution of the chain letter network depends on the age of stories (*ρ*=−0.02,*p*>0.6). However, the in-degree distribution of *Red Riding Hood* retellings (figure 7*b*) is significantly correlated with age (*ρ*=0.27,*p*<0.0001). In other words, retellings of *Red Riding Hood* do display a preference to select older versions as their pre-text(s). Note, however, that although the correlation is significant, its coefficient is rather low and the relatively low slope of the linear fit suggests that age affects in-degree only in a limited, upper-bounded range.

To account for the interaction between in-degree and age, we propose a growing network model which is based on Price's model of PA [15]. Additionally, stories have individual values of attraction which lessen over time (cf. [13,14,37]). Similar to the PA algorithm, the model begins with initializing an unconnected network consisting of *m*_0_ nodes. At each succeeding time step, a single node is added to the network. With a probability (1−*p*), a new node connects to *m*≤*m*_0_ existing nodes with uniform probability. With a probability *p*, the node is connected to *m*≤*m*_0_ nodes according to the PA mechanism. The probability to connect to an existing node is given by

4.1 $$p(i)=\frac{\alpha_{i}\beta_{i}}{\sum_{j=1}^{n}\alpha_{j}\beta_{j}},$$

where *α*_*i*_=*d*_in_(*i*) and *β*_*i*_ represents the attractiveness of a node at a particular time step. If we set *β*_*i*_ to represent a constant, the model reduces to the original PA algorithm. Following previous proposals (e.g. [14]), we propose to lessen the attractiveness of a node exponentially over time:^9^

4.2 $$\beta_{i}=\beta_{0}^{i}e^{-\tau /\gamma },$$

where $\beta_{0}^{i}$ represents a node's initial attractiveness and *τ* represents the age of a node. *γ* acts as an ‘attention span’ parameter which controls the slope of the exponential decay [37]. If *α*_*i*_ is held constant, the model's growth mechanism is restricted to the TA of nodes. Each node *i* is assigned an individual initial attractiveness value $\beta_{0}^{i}$, which is sampled from a symmetric Dirichlet distribution with hyper-parameter *ϕ*.^10^ Values of *ϕ* between zero and one generally result in a more ‘peaky’ attractiveness distribution, in which only a few nodes are highly attractive. Higher values (*ϕ*≥1) result in a more uniform attractiveness distribution. We fix *ϕ* in all experiments to 0.1. By holding the variable *α*_*i*_ and *β*_*i*_ constant, we can analyse the various types of attractiveness in isolation and their interplay. More specifically, we investigate the following four models of network growth:

(i) PA model, where *β*_*i*_=1;
(ii) temporal preferential attachment (T-PA) model, where $\beta_{0}^{i}=1$;
(iii) TA model, where *α*_*i*_=1; and
(iv) preferential attachment and temporal attractiveness (PA–TA) model.

**Figure 8.**
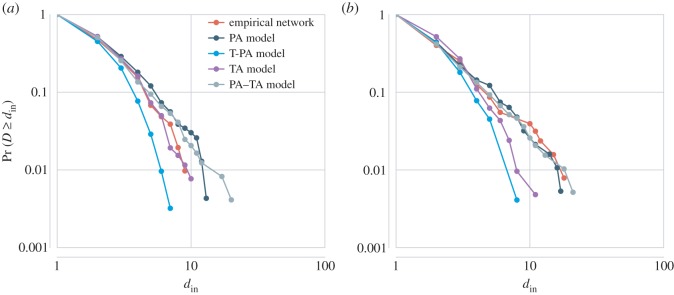
Complementary cumulative in-degree distributions of the empirically derived network and simulations of the PA, T-PA, TA and PA–TA models. Plot (*a*) shows the in-degree distribution of the chain letter network. The corresponding distributions of the four growing network models were obtained from a single simulation. Plot (*b*) provides the same information for *Red Riding Hood*.

In figure 8, we plot the complementary cumulative in-degree distributions of both story networks as well as those of the four proposed models. We compare the in-degree distributions on the basis of their corresponding Gini coefficients *G*, which are presented in table 1. The four growing network models are probabilistic and therefore results vary from simulation to simulation. The reported Gini coefficients are obtained by averaging 50 simulations.

**Table 1.** Gini coefficients of the two story networks (i.e. chain letters and *Red Riding Hood*) and the four growing network models (i.e. PA, T-PA, TA and PA–TA). The reported Gini coefficients are obtained by averaging 50 simulations for each of the four models. The empirical chain letter network displays an in-degree distribution which is most similar to those generated by the TA model. The in-degree distribution of the empirical *Red Riding Hood* network is most similar to the distributions generated by the PA–TA model.

	Gini coefficient				
	empirical	PA	T-PA	TA	PA–TA
chain letters	0.36	0.41	0.29	0.36	0.41
*Red Riding Hood*	0.43	0.41	0.29	0.37	0.43

The PA model generates heavy-tailed in-degree distributions for *Red Riding Hood*, of which the summarizing *G* values are comparable to the empirical coefficient. The Gini coefficient of the chain letter network is much smaller than those produced by the PA model. As was expected, the time-span distributions of the PA model are negatively skewed (chain letters: *median*=0.7; *Red Riding Hood*: median = 0.5) and the oldest nodes (i.e. the nodes that first enter the network) receive most of the incoming links over time. The three growing network models that take the age of nodes into account produce positively skewed time-span distributions in which younger nodes are preferred over older ones (chain letters: T-PA=0.01, TA=0.03, PA–TA=0.04; *Red Riding Hood*: T-PA=0.06, TA=0.07, PA–TA=0.08). The Gini coefficients of the T-PA model are, however, rather small compared to the empirical coefficients. The TA model generates in-degree distributions that display the most similar degrees of inequality to those of the chain letter network. In the case of *Red Riding Hood*, the best results are obtained by the PA–TA model.

## 5. Discussion

In this article, we have studied the structure and evolution of story networks. Story networks, defined as non-hierarchical agglomerations of pre-textual relationships, represent streams of retellings in which retellers modify and adapt retellings in a gradual and accumulative way. The first challenge was to develop methods that allow us to automatically extract such story networks from raw text collections. To this end, we have proposed a clustering procedure, termed *Bootstrap Neighbor Clustering*, which approaches the problem of pre-text selection as an open-set problem and attempts to bootstrap pre-textual story networks. We have constructed a story network for a large diachronic collection of Dutch *Red Riding Hood* retellings. To obtain a better understanding of the structural properties of this network, we have systematically compared it to a story network extracted from a corpus of paper chain letters.

Using these extracted networks as a base, we were able to provide a mechanistic understanding of story network growth and, by extension, of retellers’ motivations for choosing particular story versions to base their retelling on. We hypothesized that stories are differentially preferred to function as a retelling's pre-text given three types of attractiveness: frequency-based, temporal and model-based attractiveness. To gain more insight into the relationships between stories and their pre-texts, we assessed the patterns of connectivity of the two story networks by performing a rigorous statistical analysis of their in-degree distributions. The in-degree distribution of *Red Riding Hood* displays heavy-tail properties that are well characterized by means of a power-law or lognormal model. Such heavy-tailness implies that a large proportion of stories have small in-degree values and only a few, yet significant number of stories function as pre-textual context for a large number of other stories. The heavy-tail distribution of *Red Riding Hood* contrasts with the relatively uniform in-degree distribution of the chain letter network, which we characterized as exponential.

In addition, we have demonstrated that retellings of chain letters and *Red Riding Hood* are published in relatively close temporal proximity. As was to be expected, the effect of TA is strongly observed in the chain letter corpus. Its story network displays a chain-like structure that is reminiscent of the letter's request to be redistributed within a short period of time. It was shown that the time-span distribution of the chain letters does not display a positive correlation with its corresponding in-degree distribution. Retellings of *Red Riding Hood*, on the other hand, do exhibit a significant correlation between in-degree and age. These contrasting results can be linked to an important difference between the chain letter and *Red Riding Hood* retellings: whereas retellers of *Red Riding Hood* can choose from a vast amount of story versions, chain letter retellers are requested to redistribute and retell one specific version. Moreover, we have shown that, in the retelling of chain letters, the mechanism of PA has no effect.

To explore which mechanisms potentially underlie the evolution of the two story networks, we have investigated a model of network growth. In addition to a PA mechanism, this model implements a form of model-based attractiveness which decays exponentially in time. The model that incorporates both preferential attachment and temporal attractiveness (PA–TA) best simulates the in-degree distribution of *Red Riding Hood*. The more parsimonious TA model sufficed to account for the observed in-degree distribution of the chain letter network. This result concurs with the finding that the in-degree distribution of the chain letter network does not depend on age. Both models of network growth generate positively skewed time-span distributions that are comparable to the observed story network distributions.

This being said, we wish to stress that there is not one true story network. In this article, we made a rather conservative and simplifying choice by investigating pre-textual relationships between stories on the basis of their vocabulary. However, the number of dimensions on which stories can be considered (dis)similar is virtually endless. In order to avoid falling into an ‘essentialist’ trap, future research should first be directed towards studying different dimensions of story similarity (e.g. topics, motifs, genre, cf. [38]) and their effect on the structure of story networks. A second point of future research is to further explore macroscopic properties of story networks. While the present study mainly focused on the in-degree distributions of story networks, one needs to investigate other general principles governing their structure and evolution in order to obtain a more profound understanding of story networks. Many complex real-world networks can be characterized as the so-called ‘small-world networks’, which exhibit two fundamental properties: (i) locally connected groups of nodes and (ii) a short average shortest path length between nodes [20,39]. We take it to be an interesting question whether story networks display the small-world property. In this scenario, stories would be connected to only a few other stories, while at the same time all stories in the network would be connected to each other through only a few intermediate steps. Another principle governing many real-world networks is a modular structure. Networks with modular structure are hierarchically organized into local groups of densely connected nodes, with a low density of connections *between* groups [20]. A modularity analysis of story networks could reveal that stories with high in-degrees function as bridges between local ‘story communities’ and integrate them into a single network [40].

## Footnotes

1 In fact, Rhodopis’ story exhibits three of the five main characteristics attributed to the Cinderella story type (as characterized by Aarne & Thompson [2] in the folktale catalogue *The types of the folktale*): help of an animal (2), proof of identity (4) and marriage with the prince (5).

2 In evolutionary terms, such networks can be described as lineages or phylogenetic trees. We prefer to use the term network, however, because it does not presume a clear ‘root node’ from which all subsequent story versions have supposedly sprang (cf. [4,5]).

3 These types resemble the concepts of content- and context-based biases in theoretical models of cultural evolution (cf. [6]).

4 Translation of Antoniorrobles’ *Caperucita Encarnada pasó un susto … y luego ¡nada!* by Sandra Beckett [24].

5 An interactive search engine of the collection is available at http://fbkarsdorp.github.io/rrh-browser/.

6 Available from https://languagemachines.github.io/ucto/.

7 To avoid any misunderstandings, it should be stressed that we do *not* treat stories and pre-texts as two separate classes, as any story can become a pre-text to a later story.

8 These random graphs are created by randomly rewiring the edges of the story networks while preserving the time constraint, i.e. stories may only link to randomly chosen pre-texts of the same or older age.

9 Eom & Fortunato add a node's in-degree to its attractiveness at a particular time step [14]. We choose to weight a node's in-degree by its attractiveness by *multiplying* the two values.

10 Our choice for sampling from a Dirichlet distribution is largely motivated by the fact that Dirichlet processes are often employed to model data that, like the presented story networks, tend to develop in a ‘rich get richer’ fashion.

## Data accessibility

We have released our data as Folgert Karsdorp (2016). Story network datasets. Zenodo. 10.5281/zenodo.51588. The complete datasets can be downloaded from: http://dx.doi.org/10.5281/zenodo.51588.
